# Trehalose or not-trehalose: The question of direct vs. indirect transcriptional responses to the sugar trehalose-6-phosphate

**DOI:** 10.1093/plphys/kiae306

**Published:** 2024-05-31

**Authors:** Thu M Tran, Kyle W Swentowsky

**Affiliations:** Assistant Features Editor, Plant Physiology, American Society of Plant Biologists; Cold Spring Harbor Laboratory, Cold Spring Harbor, NY 11724, USA; Assistant Features Editor, Plant Physiology, American Society of Plant Biologists; Cold Spring Harbor Laboratory, Cold Spring Harbor, NY 11724, USA

In terrestrial ecosystems, plants act as primary producers by sequestering carbon from CO_2_ and incorporating it into the soluble sugar form. These sugars are transported out of the leaves, usually in the form of sucrose, and into sink organs such as roots and fruits. The distribution of sugars within the plant at any given time is non-uniform, and plants must continually monitor their sugar levels to control carbohydrate homeostasis and other signaling processes. In addition to directly sensing the primary soluble sugar, it has been proposed that plants can also perceive trehalose-6-phosphate (Tre6P) as a signal of sugar availability. Tre6P is the intermediate molecule in the synthesis of the sugar trehalose. Trehalose metabolism is facilitated by 2 enzymes: trehalose-6-phosphate synthase (TPS) and trehalose-phosphate phosphatase (TPP). In the first step, TPS catalyzes the synthesis of Tre6P from UDP-glucose and glucose-6-phosphate. TPP then dephosphorylates Tre6P to produce trehalose (Fig.).

In addition to its role in sugar sensing, Tre6P controls developmental processes such as flowering time, inflorescence architecture, and shoot branching (Satoh-Nagasawa et al. 2006; Wahl et al. 2013; Fichtner et al. 2021, reviewed in Fichtner and Lunn 2021). While it is understood that Tre6P can rapidly induce changes in gene expression, the mechanisms of Tre6P perception and downstream signaling are still elusive.

In this study, Avidan et al. (2024) used an inducible TPS enzyme to identify the direct gene expression targets of Tre6P. The authors used an ethanol-inducible version of the TPS enzyme from *E. coli* (iTPS) transformed into Arabidopsis to analyze the short-term (4 to 6 h) transcriptional responses to Tre6P. Previous studies have been limited by genetic interventions that altered TPS and TPP expression causing constitutive changes in both Tre6P and sucrose contents. The short-term inducible system allowed the researchers to capture the rapid responses before Tre6P affected sucrose synthesis, therefore partially overcoming technical issues associated with constitutively altering TPS expression. The authors compared the transcriptional output of iTPS with the response to increased sugar availability across 9 treatments from previous studies and calculated an average response, termed a carbon response factor. Transcripts that showed a similar response to iTPS and elevated sugar were considered likely targets of Tre6P signaling.

In agreement with previous studies of Tre6P-induced gene expression, elevated Tre6P led to widespread changes in transcript abundance for almost one-half of the transcriptome. Through this deconvolution process, around 40% of these transcripts are likely responses to Tre6P. This data set was used to elucidate how Tre6P affects related biological processes.

The authors then analyzed the effects of induced Tre6P on global gene expression, focusing on interactions between Tre6P and 3 well-known sugar-signaling modules: SnRK1, TORC, and S_1_ bZIP transcription factors. SUCROSE-NON-FERMENTING1-RELATED KINASE1 (SnRK1) plays a key role in low-energy signaling (Jossier et al. 2009). Previous studies suggested that Tre6P can act by inhibiting SnRK1, but there is a missing link in the evidence: the in vitro inhibition of SnRK1 by Tre6P was observed only in the extraction from sink tissues, and the change of downstream targets of SnRK1 transcripts was observed only in mature leaves (source tissues). The results presented here reveal a complex relationship between Tre6P and SnRK1-signaling modules with implications for cellular responses to sugar availability. Elevated Tre6P levels consistently and primarily inhibit the SnRK1 starvation response. Additionally, Tre6P influences the expression of SnRK1 protein subunits and the expression of its interactors, indicating a tight interplay between Tre6P signaling and SnRK1 function.

Next, the interaction of Tre6P with the TARGET OF RAPAMYCIN COMPLEX (TORC)-signaling module was considered. TORC is a canonical positive regulator of ribosome biogenesis, and SnRK1 represses TORC signaling in plants (Baena-González et al. 2007), suggesting a possible relationship between Tre6P and TORC. Interestingly, the authors found that Tre6P likely influences ribosome production through SnRK1 rather than directly impacting TORC signaling. Additionally, Tre6P affects the expression of TORC phosphorylation targets, suggesting that coordinated actions between Tre6P and TORC regulate cellular responses.

Finally, Avidan et al. (2024) investigated the interactions between Tre6P and bZIP signaling, focusing on sugar translationally regulated bZIPs (S_1_ bZIPs). Under low-sugar conditions, S_1_ bZIPs activate starvation responses (Ma et al. 2011). There is an overlapping set of transcripts between iTPS and overexpression of the S_1_ bZIP, bZIP11, and most of these transcripts showed opposite responses, suggesting that Tre6P adds another control layer, making plants more responsive to low-sugar conditions.

In summary, the results from this study improve our understanding of the complex Tre6P signaling networks that plants use to react to changes in internal metabolic status and external conditions. Tre6P coordinates with other important sugar-signaling modules, SnRK1, TORC, and S_1_ bZIP, in response to sugar availability, but the main function of Tre6P signaling is to inhibit SnRK1 activity to prevent starvation responses when sugar availability is high (Fig.). However, as mentioned by the authors, since *E. coli* TPS protein is induced in all cell types while native AtTPS1 is mainly expressed in the companion and guard cells, the iTPS-inducible system did not completely remove the complications due to the secondary changes. Therefore, a future study could develop a cell type–specific inducible system to further understand the spatial aspect of Tre6P signaling. Considering the importance of Tre6P signaling and its complex connections with other signaling modules in response to sugar availability, this study provides insight into how plants cope with carbon starvation.

**Figure.**
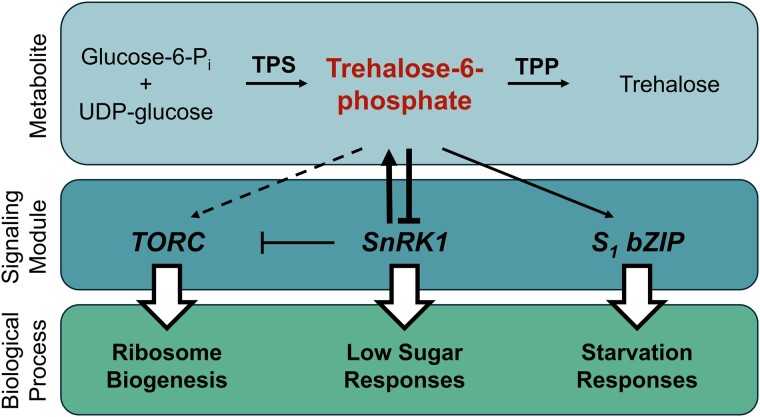
Model of relationship between trehalose-6-phosphate, signaling modules, and biological processes. Simplified metabolic pathway (top panel) resulting in trehalose synthesis. TPS catalyzes the formation of Tre6P from glucose-6-phosphate and UDP-glucose; TPP catalyzes the dephosphorylation of Tre6P to Trehalose. Tre6P influences *TORC*, *SnRK1*, and *S_1_ bZIP* signaling modules (middle panel), which control biological processes (bottom panel). Solid arrows indicate known connections, dashed arrows denote potential connections.
